# A ruptured ovarian ectopic pregnancy presenting as acute abdomen: A case report

**DOI:** 10.1016/j.ijscr.2025.111335

**Published:** 2025-04-21

**Authors:** Seblewengel Maru Wubalem, Birhanu Kassie Reta, Mihret Adane Woldemichael, Sara Alemnew Wedaj, Shemsu Abraham Hussien

**Affiliations:** a Department of Pathology, Wachemo University, Hossana, Ethiopia; b Department of Pathology, Aksum University, Aksum, Ethiopia; c Department of ENT surgery, Addis Ababa University, Addis Ababa, Ethiopia; d Department of Gynecology and Obstetrics, Wachemo University, Hossana, Ethiopia

**Keywords:** Ovarian ectopic pregnancy, Acute abdomen, Ovary, Ruptured ectopic pregnancy

## Abstract

**Introduction:**

Ovarian ectopic pregnancy (OEP) is a well-known but rare type of ectopic pregnancy, accounting for approximately 0.5 %–3 % of all ectopic pregnancies. It is a potentially fatal type of ectopic pregnancy. Preoperatively diagnosis is difficult, as it can mimic tubal pregnancy or complicated ovarian cysts. The aim of this paper is to highlight the possibility of OEP in females presenting with acute abdomen and the necessity of prompt diagnosis and management of patients.

**Case presentation:**

A 28-year-old multiparous mother who had been amenorrheic for 4 weeks presents with a complaint of abdominal pain lasting 4 days. She had diffuse abdominal and cervical motion tenderness. The investigations revealed moderate anemia and right complex adnexal mass with hemoperitoneum. Right salphingoophrectomy was done for an assessment of ruptured ectopic pregnancy. Histopathologic examination confirmed ovarian ectopic pregnancy.

**Discussion:**

The incidence of ovarian pregnancy is estimated to be 1 in 7000 to 40,000 live births. It is among the causes of first-trimester maternal death. The use of intrauterine devices (IUDs) and assisted reproductive technologies (ART) are among the proposed mechanisms of OEP. The diagnosis is usually made postoperatively and confirmed by histopathological examination.

**Conclusion:**

OEP is a rare potentially fatal condition. It is a diagnostic challenge since it is rare and mimics tubal ectopic pregnancy and complicated ovarian cysts. It should always be considered in patients presenting with amenorrhea, vaginal bleeding, and abdominal pain, particularly in those using an IUD. Surgery is the mainstay of management, and histopathologic examination is crucial for confirming the diagnosis.

## Highlights

• Ovarian ectopic pregnancy is hard to diagnose due to its rarity and similarity to tubal ectopic pregnancy and complex ovarian cysts.
• It should always be considered in patients presenting with amenorrhea, vaginal bleeding, and abdominal pain.
• Surgery is the mainstay of management, and histopathologic examination is crucial for confirming the diagnosis.

## 1 Introduction

Ovarian ectopic pregnancy (OEP) is a well-known but rare type of ectopic pregnancy, accounting for approximately 0.5 %–3 % of all ectopic pregnancies [1]. A systematic review of reports on OEP published on February 2023 found 667 articles of OEP since 1945 [2]. It is a potentially fatal type of ectopic pregnancy due to its occurrence in highly vascularized ovarian tissue and its association with a higher chance of rupture of the gestational sac, which leads to intraperitoneal bleeding and hemorrhagic shock [1,3]. It is difficult to diagnose OEP preoperatively, as it can mimic tubal pregnancy or complicated ovarian cysts, both clinically and in radiological studies [1,4,5]. Here, we present a rare case of ovarian ectopic pregnancy in a 28-year-old multiparous woman. This work has been reported in line with the SCARE guideline [6].

## 2 Case presentation

A 28-year-old gravida 5 para 4 (all alive and spontaneous vaginal deliveries) woman who had been amenorrheic for 4 weeks presented to the gynecologic emergency department with a complaint of abdominal pain lasting 4 days. The pain initially was on the right side and then progressively involved the whole abdomen. Otherwise, she had no history of vaginal bleeding, contraceptive use, or trauma to the abdomen.

On physical examination, she was tachycardic (pulse rate = 120, blood pressure = 120/68, and respiratory rate = 20). She exhibited conjunctival pallor. On abdominal examination, there was diffuse abdominal tenderness with no mass or organomegaly. There was cervical motion tenderness, and the cervix was closed on per vaginum examination. Her complete blood count profile showed moderate anemia (hemoglobin = 8.9 g/dl, hematocrit = 19.6 %). The urine β-HCG was positive. Abdominal-pelvic ultrasound revealed free fluid collection in the cul-de-sac, paracolic gutter, and Morrison's pouch. The uterus was empty, and there was a 2 × 3 cm complex right adnexal mass.

Given these finding; she was taken to the operating room for an emergency exploratory laparotomy. The abdomen was entered through a midline infraumbilical incision, revealing a ruptured right ovarian ectopic pregnancy and hemolyzed hemoperitoneum, with normal-appearing contralateral adnexa. Therefore, a right salpingo-oophorectomy was performed, and 500 ml of hemoperitoneum was aspirated.

**Fig. 1:**
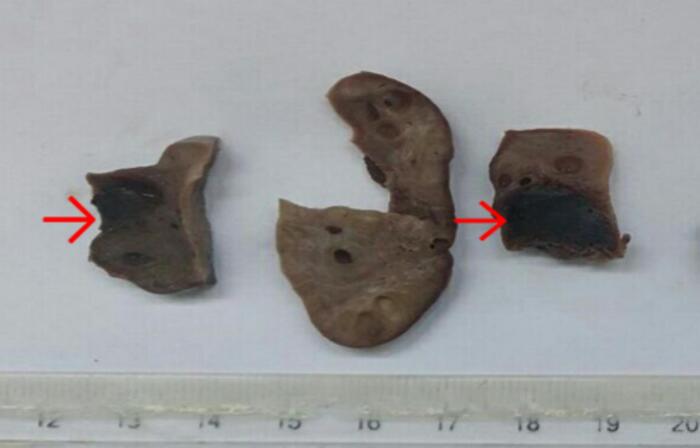
Gross image of an ovary and a hemorrhagic focus (red arrow) of an OEP. (For interpretation of the references to colour in this figure legend, the reader is referred to the web version of this article.)

**Fig. 2:**
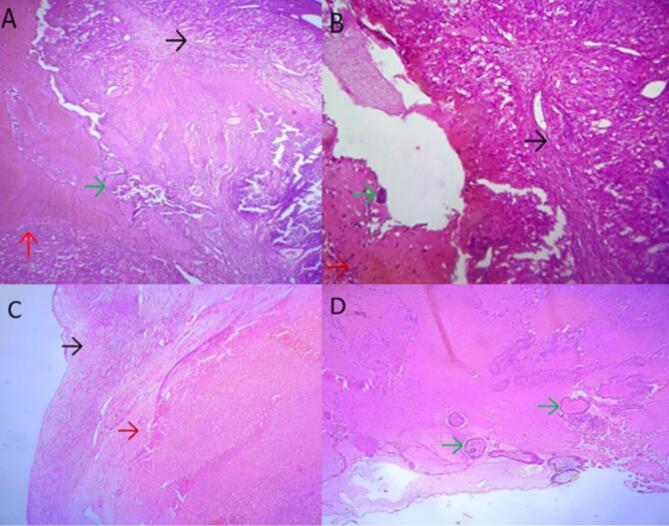
Demonstrates histopathologic images of OEP. (A) Chronic villi (green arrow) embedded in hemorrhage (red arrow) and surrounded by corpus luteum (black arrow). (B) Corpus luteum (black arrow) with hemorrhagic area (red arrow) and syncytiotrophoblast (green arrow). (C) Ovarian stroma (black arrow) and hemorrhagic focus (red arrow). (D) Chorionic villi (green arrows). embedded in hemorrhage (4×, hematoxylin and eosin). (For interpretation of the references to colour in this figure legend, the reader is referred to the web version of this article.)

The resected specimen was sent to the pathology department. Macroscopic examination demonstrated a 4.5 × 3 × 2 cm gray-white to gray-brown ovary, with a 2 × 2 cm hemorrhagic focus on cut sections (Fig. 1). Histopathologic sections revealed chorionic villi and trophoblastic cells along with hemorrhage and fibrin within the ovary, adjacent to a mature corpus luteum, confirming intraovarian pregnancy (Fig. 2).

The patient had a smooth postoperative period and was discharged on the fifth day with iron supplements and injectable contraceptives.

## 3 Discussion

The incidence of ovarian pregnancy is estimated to be 1 in 7000 to 40,000 live births [2]. Ectopic pregnancy is among the causes of first-trimester maternal death, accounting for around 10 % of pregnancy-related deaths [7].

The cause of ovarian ectopic pregnancy (OEP) is not clearly established, but several factors are associated with it. The proposed risk factors for OEP include the use of intrauterine devices (IUDs), assisted reproductive technologies (ART), endometriosis, pelvic inflammatory diseases, and previous pelvic or abdominal surgeries [2,3]. Previous pelvic or abdominal surgeries and IUDs are among the most frequently encountered risk factors, with around 81 % of OEP associated with IUDs [1,2,18]. IUDs can damage the fallopian tubal epithelium and interrupt the normal migration of the ovum [8,9]. Pelvic adhesions caused by pelvic inflammatory disease (PID), previous surgeries, or endometriosis can prevent the release of the ovum, which leads to fertilization in the ovary [3]. Various theories have been proposed regarding the mechanism of ovarian implantation in women undergoing ART, such as: uterine contractions resulting from difficult embryo transfer can lead to the retrograde movement of embryos through the fallopian tubes and into the ovary; ovarian trauma after oocyte retrieval can facilitate embryo implantation at the injured site; and a large number of spermatozoa that reach the ovary following intrauterine insemination can fertilize the ovum [3]. The patients can also present without any risk factors [10], like in this case.

Clinically, the patients presented with abdominal pain and amenorrhea, followed by vaginal bleedin [11]. When complicated by rupture, the main presentation will be hemorrhagic shock. Most of the cases are found in the first trimester [12]. However, there are reports of cases that lasted until full term [13].

The diagnosis of OEP is usually made postoperatively and confirmed by histopathological examination. Its similar clinical presentation and sonographic features to tubal pregnancy and complicated ovarian cysts make preoperative diagnosis difficult. The diagnosis remains challenging even during surgery [14,15]. Therefore; Spiegelberg's criteria are used for intraoperative diagnosis. The criteria include the presence of a normal ipsilateral tube that is separate from the ovary, the pregnancy occupying a normal position on the ovary, the ovary being attached to the uterus by the ovarian ligament, and pathologic confirmation of placental tissue attached to the ovarian stroma [9,15]. Ultrasound is the preferred imaging modality for the diagnosis of OEP. The classic sonographic features are a cyst with a wide echogenic outer ring and an empty uterus [8,15,18]. In this patient, the abdominopelvic ultrasound shows an empty uterus and a right adnexal complex mass, but it does not definitively indicate the exact location of the ectopic pregnancy. Therefore, the diagnosis of OEP was made intraoperatively and confirmed by histopathologic examination.

Surgery is the main modality of treatment for OEP, either laparotomy or laparoscopic surgery [1]. Laparoscopic surgery is becoming the preferred treatment over laparotomy since it has fewer complications and a shorter hospital stay [1,5,16]. Medical treatment with methotrexate (MTX) is controversial. There are reports of successful management of OEP with MTX [17]. However, Xin Zhou et al. report a trial of MTX treatment in three patients, which failed in all cases, and they were later managed surgically [1]. Our patient has been managed surgically by laparotomy since laparoscopic surgery is not available in our facility.

## 4 Conclusion

In conclusion, OEP is a rare potentially fatal condition. It is a diagnostic challenge since it is rare and mimics tubal ectopic pregnancy and complicated ovarian cysts. It should always be considered in patients presenting with amenorrhea, vaginal bleeding, and abdominal pain, particularly in those using an IUDs. Surgery is the mainstay of management, and histopathologic examination is crucial for confirming the diagnosis.

## Abbreviations

ART: assisted reproductive technologies
IUDs: intrauterine devices
OEP: Ovarian ectopic pregnancy
PID: pelvic inflammatory disease
MTX: methotrexate
β- HCG: β- human chorionic gonadotropin

## Consent for publication

Written informed consent was obtained from the patient for publication of this case report and accompanying images.

## Ethical approval

The study was notified to the university ethics committee; but this is case report and it does not need a specific ethical approval.

## Guarantor

Seblewengel Maru Wubalem

Shemsu Abraham Hussien

## Funding

This work did not receive any specific grant from funding agencies in the public, commercial, or not-for-profit sectors*.*

## Author contribution

1. Seblewengel Maru Wubalem: Conceptualization; Data curation; Resources; Visualization; riting – original draft; Writing – review and editing
2. Birhanu Kassie Reta: Data curation; Visualization; Writing – review and editing
3. Mihiret Adane Woldemichael: Data curation; Visualization; Writing – review and editing
4. Sara Alemnew Wedaj: Data curation; Visualization; Writing – review and editing
5. Shemsu Abraham Hussien: Data curation; Supervision; Visualization; Writing – original draft; Writing – review and editing

## Declaration of competing interest

The authors declare that they have no known competing financial interests or personal relationships that could have appeared to influence the work reported in this paper.
